# Heat as a Remedial Agent

**Published:** 1892-09

**Authors:** 


### HEAT AS A REMEDIAL AGENT.

Eczema, moist tetter, or salt-rheum, is one of the most troublesome of skin infections, not infrequently defying skillful medical treatment for years. Sufferers from this affection will be glad to know that one of the best means of relieving the intolerable itching which accompanies it is a simple remedy which is always accessible, namely, the application of heat. Hot water applied at a temperature as high as can be borne without actual injury to the skin, is an almost certain remedy to relieve the intolerable itching. The parts should never be scratched or rubbed so as to increase the irritation. Simply holding the affected part near the fire of an open grate, gradually approaching more and more close until the degree of heat becomes almost painful, is another means of applying the same remedy.

Again, there is no better remedy for the relief of rheumatic pains in the joints or other portions of the body, than hot applications. Flannel cloths dipped in very hot water and wrung as dry as possible should be applied to the parts, and the whole enveloped in a thick, dry flannel cloth to retain the heat. The application should be renewed every five or ten minutes. The application of ground mustard in the proportion of a tablespoonful to the quart of water, increases the effect of the heat. A teaspoonful of turpentine sprinkled upon the fomentation just before it is applied, or a cloth saturated with a solution of one part turpentine to two or three of alcohol, applied over the affected part and covered by the fomentation, is also a means of intensifying the effect of the fomentation.

The various liniments used for rheumatism have little or no curative value, although some are useful for the relief of pain. One of the best is a simple preparation consisting of equal parts of olive oil and oil of wintergreen. It should be applied carefully, however, as the pure oil of wintergreen is quite a vigorous irritant. Menthol liniment is also a useful application.

